# Prognostic Implications and Immune Infiltration Analysis of ALDOA in Lung Adenocarcinoma

**DOI:** 10.3389/fgene.2021.721021

**Published:** 2021-12-03

**Authors:** Guojun Lu, Wen Shi, Yu Zhang

**Affiliations:** Department of Respiratory Medicine, Nanjing Chest Hospital, Affiliated Nanjing Brain Hospital, Nanjing Medical University, Nanjing, China

**Keywords:** lung adenocarcinoma, AldoA, biomarker, prognosis, immune infiltration

## Abstract

**Background:** aldolase A (*ALDOA*) has been reported to be involved in kinds of cancers. However, the role of *ALDOA* in lung adenocarcinoma has not been fully elucidated. In this study, we explored the prognostic value and correlation with immune infiltration of *ALDOA* in lung adenocarcinoma.

**Methods:** The expression of *ALDOA* was analyzed with the Oncomine database, the Cancer Genome Atlas (TCGA), and the Human Protein Atlas (HPA). Mann-Whitney *U* test was performed to examine the relationship between clinicopathological characteristics and *ALDOA* expression. The receiver operating characteristic (ROC) curve and Kaplan-Meier method were conducted to describe the diagnostic and prognostic importance of *ALDOA*. The Search Tool for the Retrieval of Interacting Genes (STRING) and Cytoscape were used to construct PPI networks and identify hub genes. Functional annotations and immune infiltration were conducted.

**Results:** The mRNA and protein expression of *ALDOA* were higher in lung adenocarcinoma than those in normal tissues. The overexpression of *ALDOA* was significantly correlated with the high T stage, N stage, M stage, and TNM stage. Kaplan-Meier showed that high expression of *ALDOA* was correlated with short overall survival (38.9 vs 72.5 months, *p* < 0.001). Multivariate analysis revealed that *ALDOA* (HR 1.435, 95%CI, 1.013–2.032, *p* = 0.042) was an independent poor prognostic factor for overall survival. Functional enrichment analysis showed that positively co-expressed genes of *ALDOA* were involved in the biological progress of mitochondrial translation, mitochondrial translational elongation, and negative regulation of cell cycle progression. KEGG pathway analysis showed enrichment function in carbon metabolism, the HIF-1 signaling pathway, and glycolysis/gluconeogenesis. The “SCNA” module analysis indicated that the copy number alterations of *ALDOA* were correlated with three immune cell infiltration levels, including B cells, CD8^+^ T cells, and CD4^+^ T cells. The “Gene” module analysis indicated that *ALDOA* gene expression was negatively correlated with infiltrating levels of B cells, CD8^+^ T cells, CD4^+^ T cells, and macrophages.

**Conclusion:** Our study suggested that upregulated *ALDOA* was significantly correlated with tumor progression, poor survival, and immune infiltrations in lung adenocarcinoma. These results suggest that *ALDOA* is a potential prognostic biomarker and therapeutic target in lung adenocarcinoma.

## Introduction

According to the latest data from global cancer statistics, lung cancer is the most commonly diagnosed cancer and the leading cause of cancer-related death around the whole world (Bray et al., 2018). Lung adenocarcinoma is the most common pathological type and accounts for more than 40% of all lung cancers (Travis et al., 2015; Denisenko et al., 2018). Despite advances that have been made in early diagnosis and treatment for lung adenocarcinoma in the past years, including targeted therapy and immunotherapy (Zhou and Yao, 2016; Hanna et al., 2017; Xu et al., 2018), the prognosis of lung adenocarcinoma patients remains bleak (Zhang et al., 2019). Therefore, it is imperative to search for novel prognostic markers and therapeutic targets for lung adenocarcinoma.

Aldolase A (*ALDOA*), also called muscle-type aldolase, is mainly expressed in muscle tissues (Tochio et al., 2010). *ALDOA* encodes a glycolytic enzyme that catalyzes the reversible conversion of fructose-1,6-bisphosphate to glyceraldehyde 3-phosphate and dihydroxyacetone phosphate. Ectopic expression of *ALDOA* is important in the development of cardiac hypertrophy, heart failure, and many cardio-cerebrovascular diseases (Hu et al., 2013). Furthermore, *ALDOA* has been reported to be involved in gluconeogenesis and glycolysis (Zeng et al., 2016). Based on both gluconeogenesis and glycolysis can provide energy for tumor proliferation, accumulating evidence has indicated that *ALDOA* plays an important role in the pathological progress of several cancers. A paper from Saito *et al.* indicated that upregulated *ALDOA* in cervical adenocarcinoma can increase the metastasis and invasion of cervical adenocarcinoma cells via promoting epithelial-mesenchymal transition (EMT) (Saito et al., 2020). Concerning non-small cell lung cancer, Fu et al. reported that *ALDOA* can activate the EGFR/MAPK pathway to promote cyclin D1 expression, enhance proliferation and G1/G transition, and facilitate aerobic glycolysis (Fu et al., 2018). These findings indicate that *ALDOA* plays an important role in tumor progression.

In the present study, we conducted bioinformatics analyses on *ALDOA* in lung adenocarcinoma patients, including transcriptional expression and mutation analysis, survival analysis, functional enrichment analysis. We also performed co-expression analysis, constructed the predicted protein-protein interaction (PPI) networks, and identified hub genes of co-expressed genes with *ALDOA*. Moreover, we determined the relationship between *ALDOA* expression and immune cell infiltration in lung adenocarcinoma. Our results link the expression of *ALDOA* with a poor prognosis and provide a potential therapeutic target for lung adenocarcinoma.

## Materials and Methods

### Oncomine Database

Oncomine (https://www.oncomine.org/) is an online platform that provides solutions to compute gene expression signatures, clusters, and gene-set modules, automatically extracting biological insights (Rhodes et al., 2007). In this study, we conducted Oncomine to evaluate the mRNA expression of *ALDOA* in lung adenocarcinoma. The results drew from a series of lung adenocarcinoma studies, including Selamat lung, Landi lung, Hou lung, Okayama lung, Stearman lung, Su lung, and Garber lung (Garber et al., 2001; Stearman et al., 2005; Su et al., 2007; Landi et al., 2008; Hou et al., 2010; Okayama et al., 2012; Selamat et al., 2012).

### The Cancer Genome Atlas (TCGA)

TCGA (https://portal.gdc.cancer.gov/) is a genomics data resource that characterized, and analyzed cancer samples (Tomczak et al., 2015). In this study, we analyzed the transcription level of *ALDOA* in multiple cancers from TCGA. The mRNA expression and associated clinical data of *ALDOA* in lung adenocarcinoma were also downloaded from TCGA. The mRNA data of FPKM format has been converted into TPM.

### Tumor Immune Estimation Resource (TIMER)

TIMER (http://timer.cistrome.org/) is an online database and allows users to analyze the differential expression between tumor and normal tissues across all TCGA tumors, and study the correlation between gene expression and immune infiltration level (Li et al., 2020). In the present study, we conducted TIMER to determine the expression of *ALDOA* in diverse cancer types. Moreover, we applied TIMER to explore the correlation between *ALDOA* expression and the abundance of tumor-infiltrating immune cells (B cells, CD4^+^ T cells, CD8^+^ T cells, neutrophils, macrophages, and dendritic cells). In addition, TIMER was used to study the correlation between *ALDOA* expression and gene markers of tumor-infiltrating immune cells.

### UALCAN

The UALCAN (http://ualcan.path.uab.edu/) is a comprehensive online web resource that provides easy access to analyze publicly available cancer omics data (Chandrashekar et al., 2017). In this study, we performed UALCAN to compare the mRNA and protein expression of *ALDOA* from TCGA and Clinical Proteomic Tumor Analysis Consortium (CPTAC, https://proteomics.cancer.gov/programs/cptac) (Edwards et al., 2015).

### The Human Protein Atlas (HPA)

The HPA database (https://proteinatlas.org/) is aimed to map all the human proteins with an integration of various omics technologies (Uhlén et al., 2015; Uhlen et al., 2017). All the data of human proteins includes expression profiles in cells, tumor tissues, and normal tissues. In this study, we performed HPA to confirm the protein expression of *ALDOA* in lung adenocarcinoma.

### Gene Expression Profiling Interactive Analysis (GEPIA2)

GEPIA 2 (http://gepia2.cancer-pku.cn/) is a web-based tool to provide interactive and customizable functions, including gene expression analysis, correlation analysis, survival analysis, similar genes detection, and dimensionality reduction analysis (Tang et al., 2019). In this study, we conducted GEPIA2 to examine the correlation between *ALDOA* expression and overall survival. In addition, GEPIA2 was used to assess the correlation between *ALDOA* expression and gene markers of tumor-infiltrating immune cells.

### The Kaplan Meier Plotter

The Kaplan Meier plotter (http://www.kmplot.com/analysis/) is an online tool to assess the effect of 54k genes on survival across cancers including breast, ovarian, lung, and gastric cancer (Nagy et al., 2021). Gene expression data and information of relapse-free and overall survival are downloaded from Gene Expression Omnibus (GEO), European Genome-phenome Archive (EGA), and TCGA. In this study, we performed a Kaplan Meier plotter to validate the prognostic value of *ALDOA* in lung adenocarcinoma.

### c-BioPortal Database

The c-Bio Cancer Genomics Portal (https://www.cbioportal.org/) is an open-access online resource for interactive exploration of many cancer genomics databases (Cerami et al., 2012). The cancer research community can utilize genomic data easily and directly with c-BioPortal. In this study, we performed c-BioPortal databases to explore mutation data of *ALDOA* in lung adenocarcinoma, obtain its prognostic value in altered lung adenocarcinoma patients, acquire co-expressed genes of *ALDOA*, and determine the correlation between *ALDOA* and mRNA expression of 10 hub genes.

### STRING Database and Cytoscape Platform

The Search Tool for the Retrieval of Interacting Genes (STRING, http://string-db.org, Version 11.0) is an online database to analyze functional enrichment and PPI networks (Szklarczyk et al., 2019). Cytoscape (Version 3.6.1) is an open-source software platform for integrating and visualizing complex networks (Shannon et al., 2003). In this study, to construct PPI networks of co-expressed genes and identify hub genes, we imported the co-expressed genes into STRING and then explored the degree scores with cytoHubba tool kits in Cytoscape.

### Statistical Analyses

Statistical analyses and visualization of expression differences were performed with R (V 3.6.3, https://www.r-project.org/) and R package ggplot2. Mann-Whitney *U* test was conducted to observe the differences between lung adenocarcinoma tissues and adjacent normal tissues. R package pROC (Robin et al., 2011) and clusterProfiler (Yu et al., 2012) were conducted to explore the diagnostic importance and functional enrichment analysis of co-expressed genes in lung adenocarcinoma.

## Results

### Expression of *ALDOA* in Lung Adenocarcinoma From Oncomine

**FIGURE 1:**
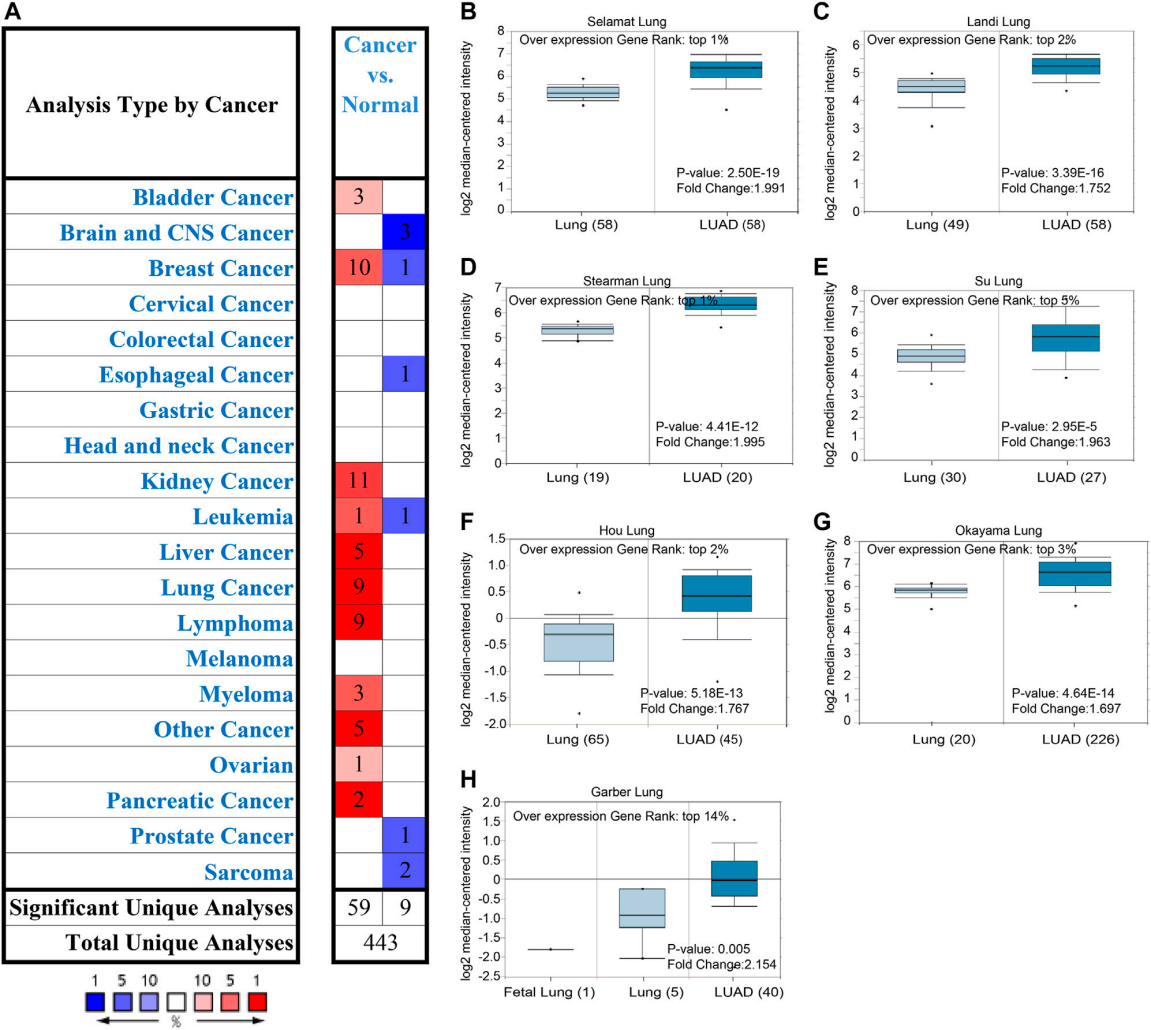
Expression of ALDOA from Oncomine **(A)** *ALDOA* expression in different types of cancers. Red means up-regulated and blue means down-regulated **(B-H)** BOX plot showing the mRNA expression levels of *ALDOA* in Selamat lung, Landi lung, Hou lung, Okayama lung, Stearman lung, Su lung, and Garber lung datasets, respectively.

To evaluate the transcription level of *ALDOA* in multiple lung adenocarcinoma studies, we performed an analysis on Oncomine. As shown in Figures 1A–H, the transcription level of *ALDOA* was upregulated in lung adenocarcinoma tissues than in normal tissues. The fold change of *ALDOA* differed from 1.697 to 2.154, and mRNA expression was up to the top 14%. These data indicated transcription level of *ALDOA* is increased in lung adenocarcinoma tissues.

### Expression of *ALDOA* in Pan-Cancer Perspective and Lung Adenocarcinoma From TCGA, UALCAN, and HPA

**FIGURE 2:**
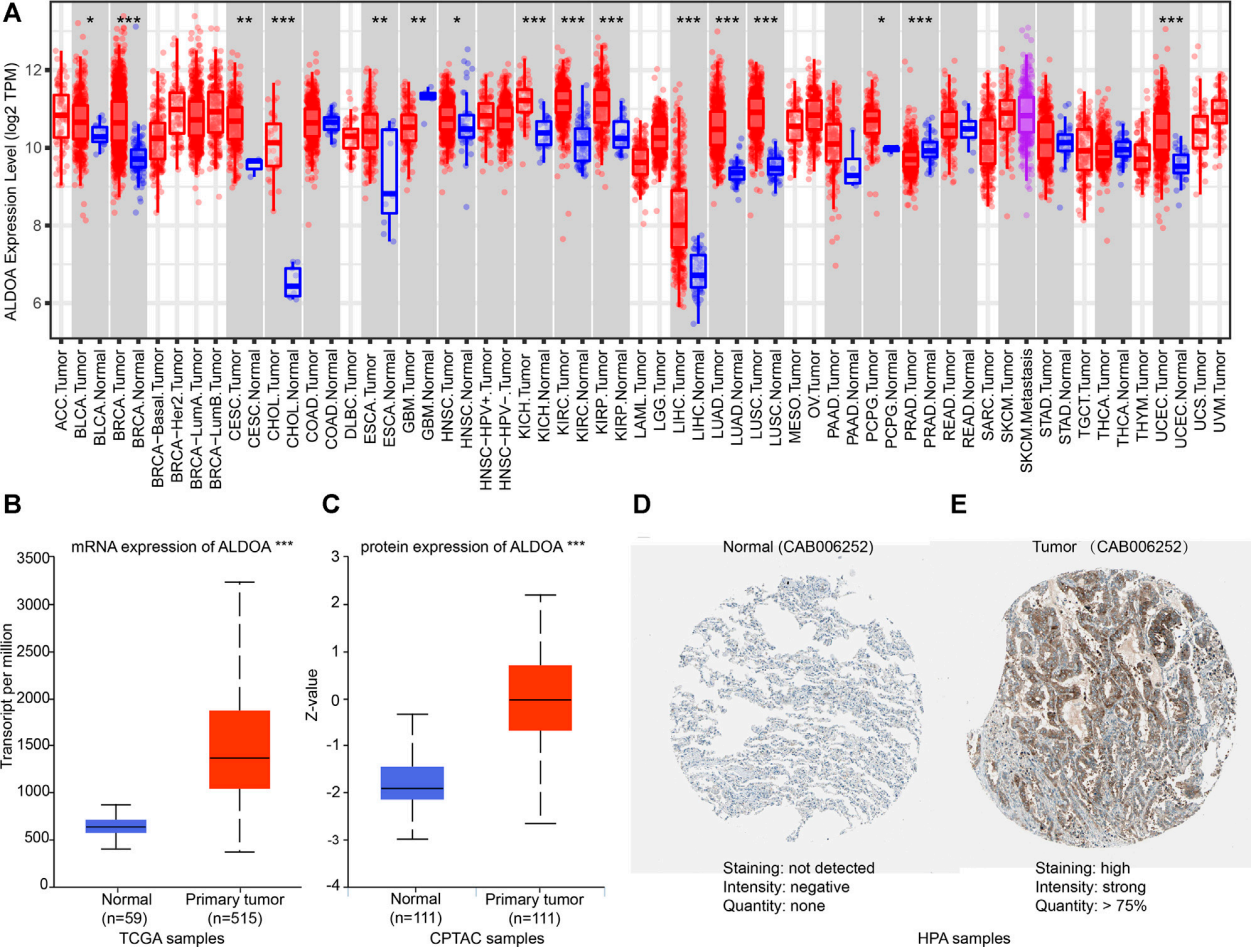
Expression of ALDOA from TCGA, CPTAC, and HPA **(A)** *ALDOA* was upregulated in 14 cancer types, including BLCA, BRCA, CESC, CHOL, ESCA, HNSC, KICH, KIRC, KIRP, LIHC, LUAD, LUSC, PCPG, and UCEC. *ALDOA* was downregulated in GBM and PRAD **(B)** The mRNA expression of *ALDOA* in 59 normal tissues and 515 lung adenocarcinoma tissues **(C)** The protein expression of *ALDOA* in 111 lung normal tissues and 111 lung adenocarcinoma tissues **(D)** The protein expression of *ALDOA* in normal tissue. Staining was not detected, intensity was negative, and quantity was none **(E)** The protein expression of *ALDOA* in lung adenocarcinoma tissue. Staining was high, intensity was strong, and quantity >75%. HPA normal: https://www.proteinatlas.org/ENSG00000149925-ALDOA/tissue/lung#img,HPA tumor: https://www.proteinatlas.org/ENSG00000149925-ALDOA/pathology/lung+cancer#img (*, *p* < 0.05; **, *p* < 0.01, ***, *p* < 0.001).

To further evaluate the expression of *ALDOA* in multiple cancers, we performed an analysis on TCGA with TIMER. As shown in Figure 2A, the mRNA expression of *ALDOA* was upregulated in 14 cancer types, including lung adenocarcinoma and lung squamous cell carcinoma. *ALDOA* was downregulated in two cancer types, including GBM and PRAD. The result from UALCAN indicated that both mRNA and protein expression of *ALDOA* in lung adenocarcinoma tissues were significantly higher than that in normal tissues (*p* = 1.62E-12, *p* = 7.75E-31, respectively) (Figures 2B,C). Consistent with the results of UALCAN, HPA showed protein expression of *ALDOA* in lung adenocarcinoma tissue was higher than that in normal lung tissue (Figures 2D,E). All these results indicate that *ALDOA* is upregulated in lung adenocarcinoma tissues.

### The Relationship Between Clinicopathological Characteristics and *ALDOA* Expression in Lung Adenocarcinoma Patients From TCGA

**FIGURE 3:**
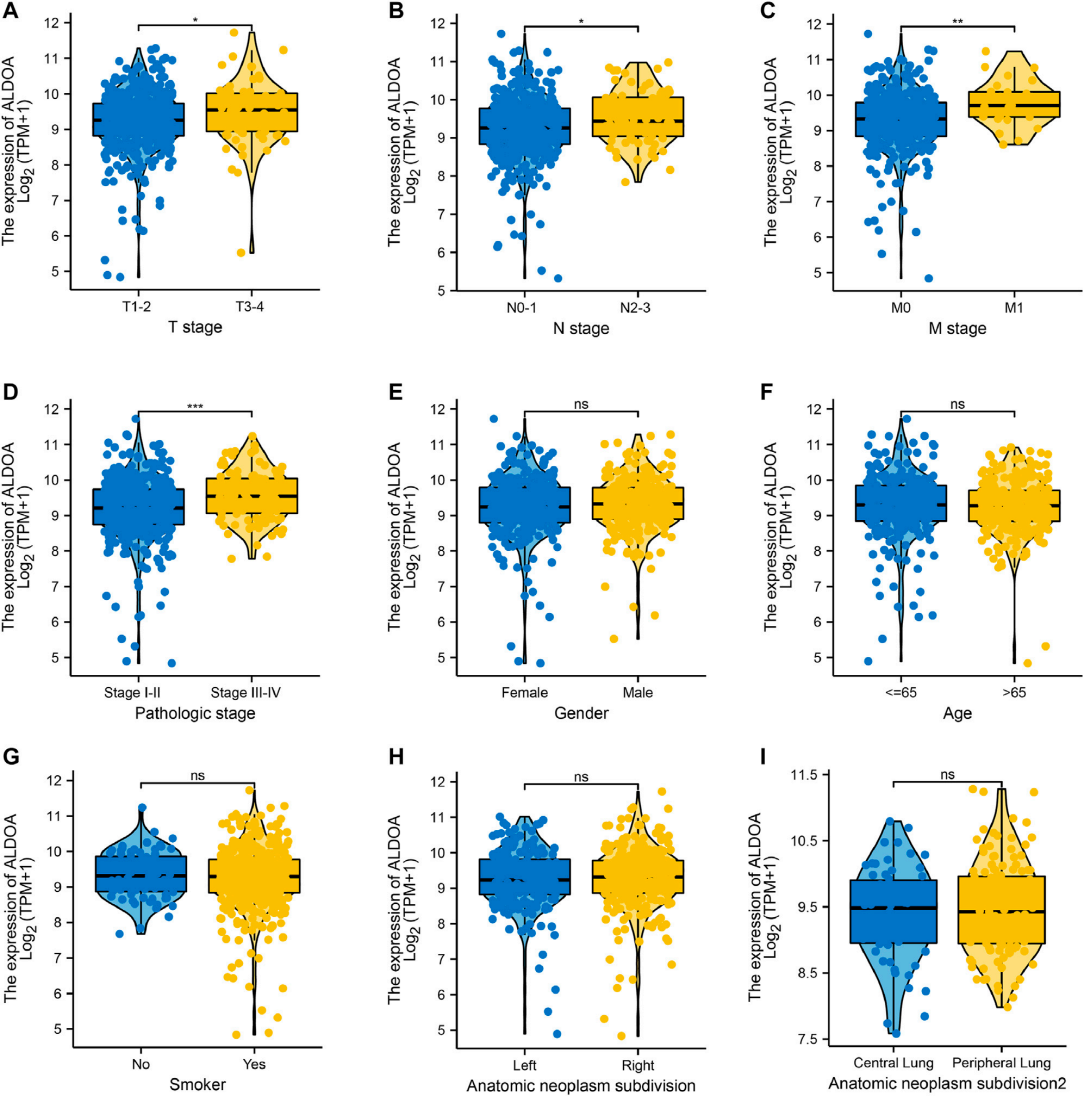
The relationship between clinicopathological characteristics and ALDOA expression in lung adenocarcinoma patients from TCGA **(A-D)** *ALDOA* mRNA expression was significantly increased with high T stage, N stage, M stage, and TNM stage **(E-I)** No significant differences were found in gender, age, smoke condition, and anatomic subdivision (right vs left, central vs peripheral) (ns, no significant; *, *p* < 0.05; **, *p* < 0.01, ***, *p* < 0.001).

We downloaded the mRNA expression and associated clinical data of *ALDOA* in lung adenocarcinoma from TCGA. The clinicopathological characteristics of lung adenocarcinoma patients were shown in Table 1. To examine the relationship between clinicopathological characteristics and *ALDOA* expression in TCGA cohorts, we conducted the Mann-Whitney *U* test. As shown in Figure 3, *ALDOA* mRNA expression in lung adenocarcinoma patients was significantly increased with high T stage (*p* = 0.025), N stage (*p* = 0.013), M stage (*p* = 0.002), and TNM stage (*p* < 0.001). However, no significantly differences were found between *ALDOA* mRNA expression and other characteristics, such as gender (*p* = 0.329), age (*p* = 0.594), smoke condition (*p* = 0.754), and anatomic subdivision (right vs left, *p* = 0.456; central vs peripheral, *p* = 0.682). To sum up, these data suggest that *ALDOA* might play an important role in tumorigenesis and metastasis of lung adenocarcinoma.

**TABLE 1:** The clinicopathological characteristics of lung adenocarcinoma patients.

Characteristics	Total	Low expression	High expression	*p*-value
	N (%)	N (%)	N (%)	
T stage	—	—	—	0.041*
** **T1	175 (32.9)	101 (19.0)	74 (13.9)	—
** **T2	289 (54.3)	136 (25.6)	153 (28.8)	—
** **T3	49 (9.2)	22 (4.1)	27 (5.1)	—
** **T4	19 (3.6)	6 (1.1)	13 (2.4)	—
N stage	—	—	—	<0.001***
** **N0	348 (67.0)	194 (37.4)	154 (29.7)	—
** **N1	95 (18.3)	31 (6.0)	64 (12.3)	—
** **N2	74 (14.3)	30 (5.8)	44 (8.5)	—
** **N3	2 (0.4)	0 (0)	2 (0.4)	—
M stage	—	—	—	0.003**
** **M0	361 (93.5)	175 (45.3)	186 (48.2)	—
** **M1	25 (6.5)	4 (1.0)	21 (5.4)	—
Pathologic stage	—	—	—	<0.001***
** **Stage I	294 (55.8)	172 (32.6)	122 (23.1)	—
** **Stage II	123 (23.3)	50 (9.5)	73 (13.9)	—
** **Stage III	84 (16.0)	33 (6.3)	51 (9.7)	—
** **Stage IV	26 (4.9)	5 (0.9)	21 (4.0)	—
Gender	—	—	—	0.178
** **Female	286 (53.5)	151 (28.2)	135 (25.2)	—
** **Male	249 (46.5)	116 (21.7)	133 (24.9)	—
Age	—	—	—	0.860
** **≤65	255 (49.4)	126 (24.4)	129 (25)	—
** **>65	261 (50.6)	132 (25.6)	129 (25)	—
Smoker	—	—	—	1.000
** **No	75 (14.4)	37 (7.1)	38 (7.3)	—
** **Yes	446 (85.6)	223 (42.8)	223 (42.8)	—
Anatomic neoplasm subdivision	—	—	—	0.282
** **Left	205 (39.4)	109 (21)	96 (18.5)	—
** **Right	315 (60.6)	151 (29)	164 (31.5)	—
Anatomic neoplasm subdivision2	—	—	—	0.816
** **Central Lung	62 (32.8)	25 (13.2)	37 (19.6)	—
** **Peripheral Lung	127 (67.2)	55 (29.1)	72 (38.1)	—

*p < 0.05; **p < 0.01; *** *p* < 0.001.

CI, confidence interval.

### Diagnostic Value of *ALDOA* for Distinguishing Lung Adenocarcinoma Tissues From Normal Tissues

**FIGURE 4:**
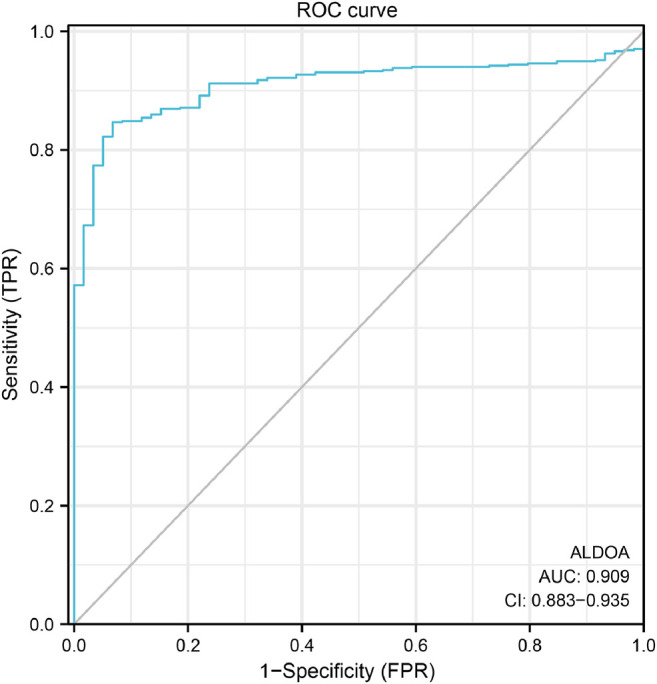
ROC curve for distinguishing lung adenocarcinoma tissues from normal tissues. With a cutoff of 8.598, *ALDOA* had a sensitivity, specificity, and accuracy of 84.7, 93.2, and 85.5%, respectively.

To study the diagnostic value of *ALDOA* for distinguishing adenocarcinoma tissues from normal tissues, we performed ROC curve analysis with R package pROC. As shown in Figure 4. *ALDOA* had an AUC value of 0.909 (95% CI: 0.883–0.935). With a cutoff of 8.598, *ALDOA* had a sensitivity, specificity, and accuracy of 84.7, 93.2, and 85.5%, respectively. This result indicates that *ALDOA* might be used as a diagnostic biomarker for distinguishing lung adenocarcinoma tissues from normal tissues.

### Correlation Between *ALDOA* Expression and Overall Survival

**FIGURE 5:**
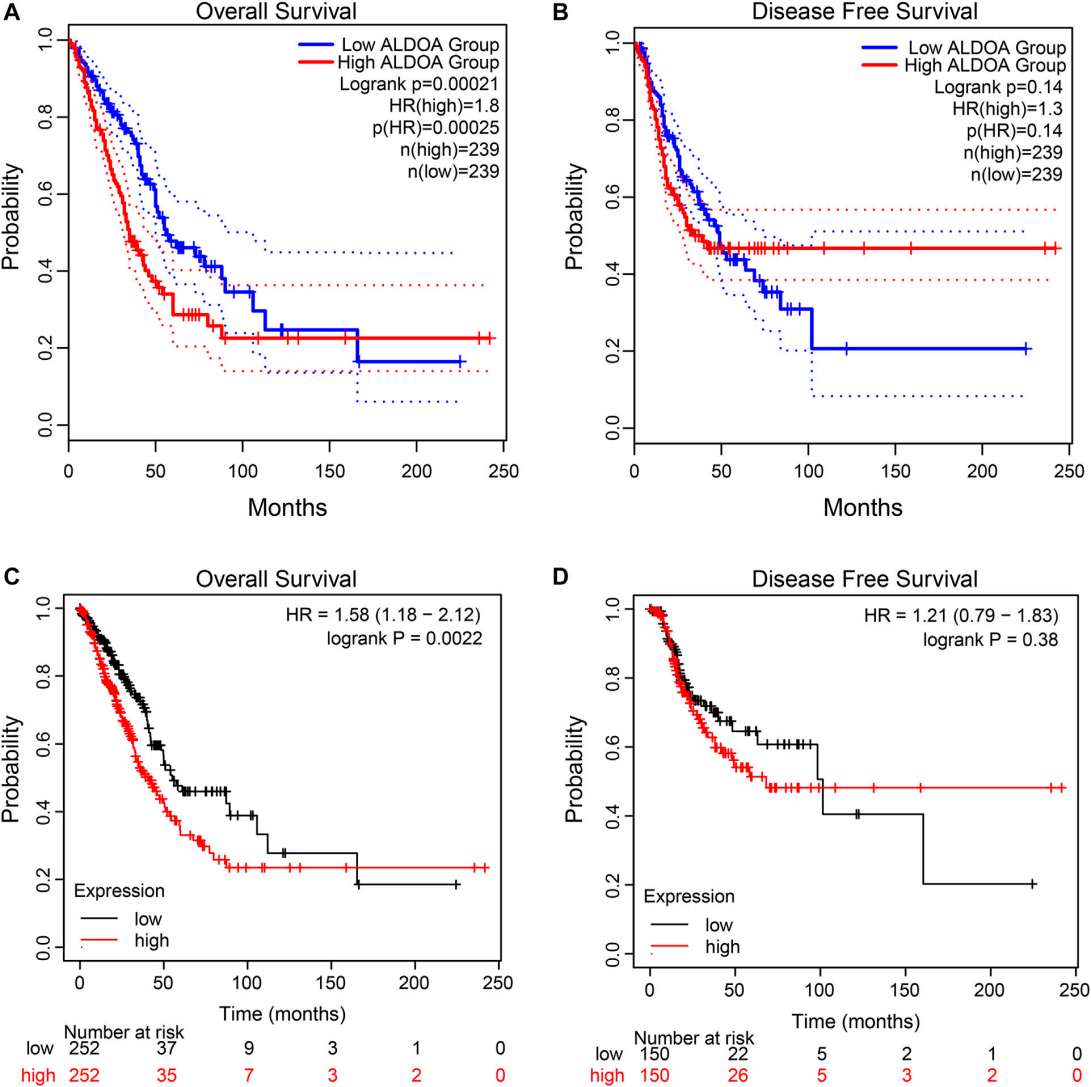
Kaplan-Meier curves for ALDOA in lung adenocarcinoma **(A)** Higher ALDOA led to shorter overall survival from GEPIA2 **(B)** ALDOA had no correlation with disease-free survival from GEPIA2 **(C)** High ALDOA was correlated with short overall survival from the Kaplan Meier plotter **(D)** ALDOA had no correlated with recurrence-free survival from the Kaplan Meier plotter.

To explore the correlation between *ALDOA* expression and overall survival and disease-free survival in lung adenocarcinoma patients, the GEPIA2 server and Kaplan Meier plotter were performed. As shown in Figures 5A,B, the GEPIA2 server indicated that the overall survival rate of lung adenocarcinoma patients with high *ALDOA* expression was significantly lower than that of patients with low *ALDOA* expression (*p* = 0.00021). However, the correlation of *ALDOA* expression with disease-free survival rate was not statistically significant (Figure 5B). Consistently, the Kaplan Meier plotter showed that lung adenocarcinoma patients with high *ALDOA* were correlated with short overall survival (41.0 vs 55.1 months, *p* = 0.0022) compared to low *ALDOA* mRNA expression (Figure 5C). There was no statistically significant between high/low expression of *ALDOA* for recurrence-free survival (68.2 vs 101.5 months, *p* = 0.38) (Figure 5D). These data indicated that high mRNA expression of *ALDOA* is correlated with short overall survival in lung adenocarcinoma.

### Prognostic Importance of *ALDOA* mRNA in Lung Adenocarcinoma Patients

**FIGURE 6:**
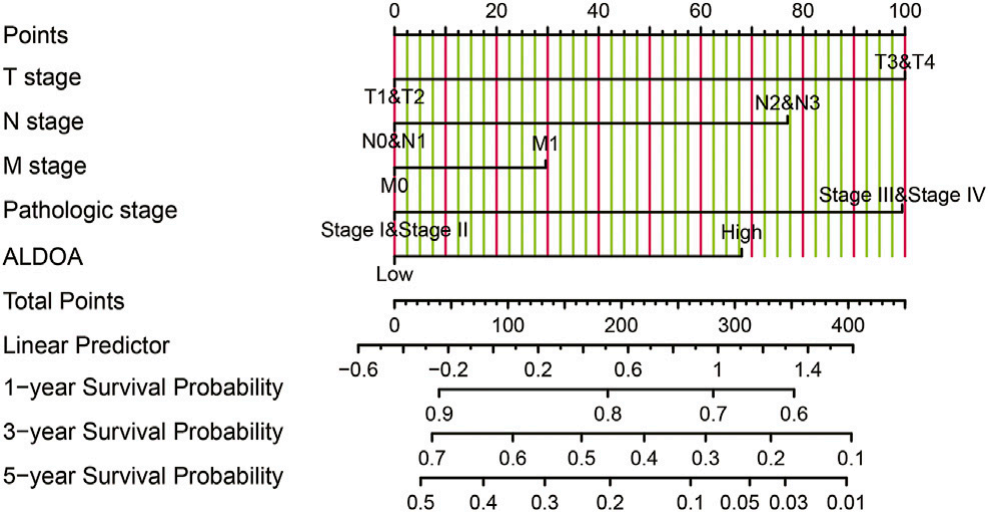
The nomogram to predict 1-, 3- and 5-years overall survival probability.

To further determine the prognostic importance of *ALDOA* mRNA, we conducted univariate and multivariate analyses with R package survival. Univariate analysis in Table 2 showed that the overall survival of lung adenocarcinoma patients was correlated with T stage, N stage, M stage, TNM stage, as well as the mRNA expression of *ALDOA* ((HR 1.799, 95%CI, 1.342–2.413, *p* = 0.011). Furthermore, we performed a multivariate analysis of five prognostic factors with the Cox proportional hazards model. As shown in Table 2, multivariate analysis revealed that T stage (HR 1.652, 95%CI, 1.020–2.673, *p* = 0.041), and mRNA expression of *ALDOA* (HR 1.435, 95%CI, 1.013–2.032, *p* = 0.042) were independent poor prognostic factors for overall survival. Moreover, a nomogram was constructed to predict the 1-, 3-, and 5-years survival probability of lung adenocarcinoma patients by combining the mRNA expression of *ALDOA* and clinical characteristics (Figure 6). Our data reveal that *ALDOA* is an independent poor prognostic factor for lung adenocarcinoma.

**TABLE 2:** Univariate and multivariate analyses of prognostic variables for overall survival.

Characteristics	Total(N)	Univariate analysis		Multivariate analysis	
		Hazard ratio (95% CI)	*p* Value	Hazard ratio (95% CI)	*p* Value
T stage (T3-4 vs T1-2)	523	2.317 (1.591–3.375)	<0.001^***^	1.652 (1.020–2.673)	0.041^*^
N stage (N2-3 vs N0-1)	510	2.321 (1.631–3.303)	<0.001^***^	1.476 (0.725–3.003)	0.283
M stage (M1 vs M0)	377	2.136 (1.248–3.653)	0.006^**^	1.158 (0.531–2.526)	0.712
Pathologic stage (Stage III- IV vs Stage I- II)	518	2.664 (1.960–3.621)	<0.001^***^	1.651 (0.771–3.534)	0.197
Gender (Male vs Female)	526	1.070 (0.803–1.426)	0.642	—	—
Age (>65 vs ≤65)	516	1.223 (0.916–1.635)	0.172	—	—
Smoker (Yes vs No)	512	0.894 (0.592–1.348)	0.591	—	—
ALDOA (High vs Low)	526	1.799 (1.342–2.413)	<0.001^***^	1.435 (1.013–2.032)	0.042^*^

*p* < 0.05; *p* < 0.01; *p* < 0.001.

### Genetic Mutation of *ALDOA* and Its Correlation With Poor Survival

**FIGURE 7:**
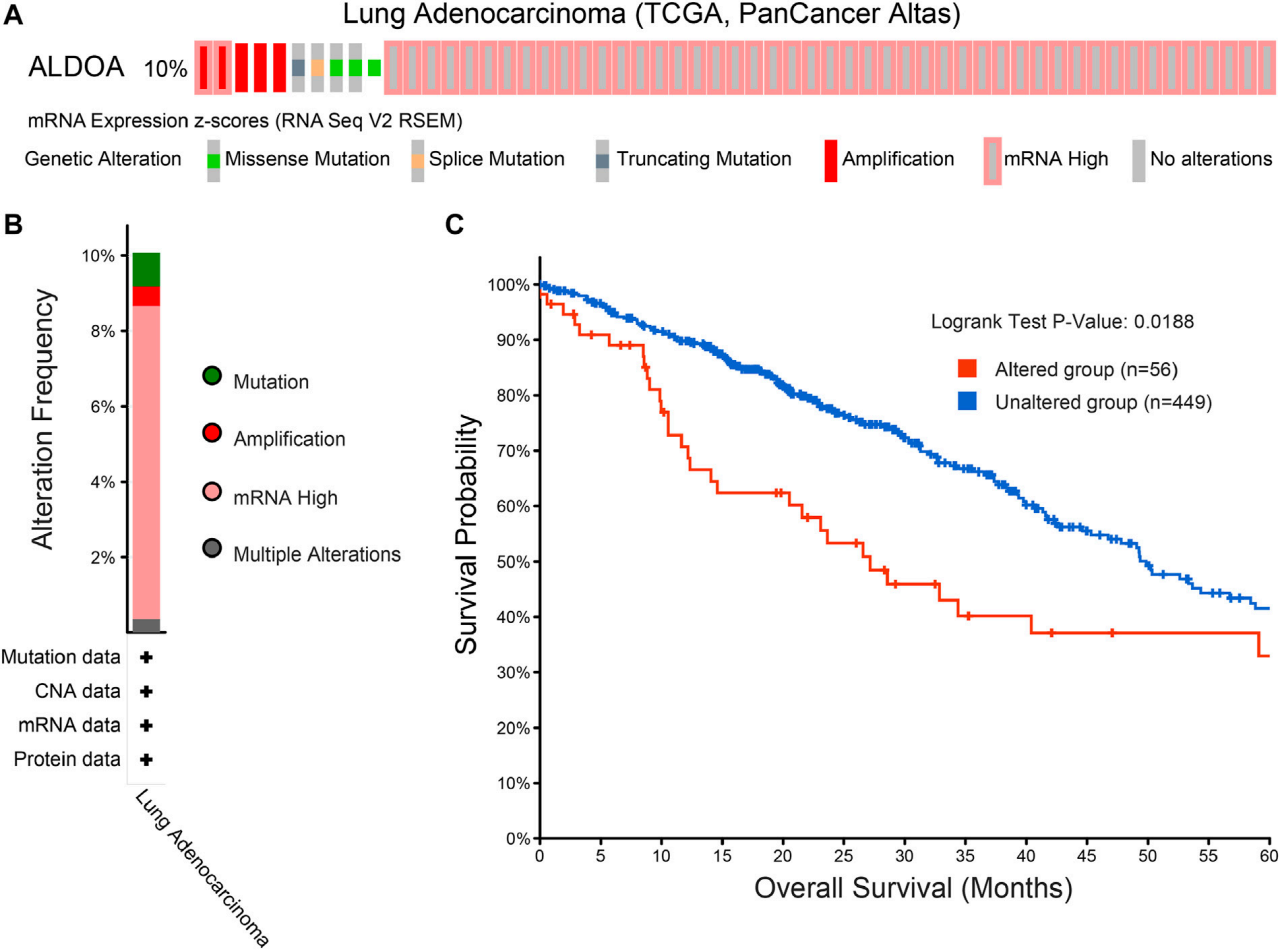
Genomic mutation of ALDOA in lung adenocarcinoma **(A)** OncoPrint of c-BioPortal showed the different mutation types and proportions of *ALDOA* **(B)** Cancer types summary showed the genomic alteration types in lung adenocarcinoma **(C)** Genomic mutation of *ALDOA* was correlated with poor overall survival in lung adenocarcinoma.

To determine the mutation characteristics of *ALDOA* and its correlation with survival in lung adenocarcinoma, we performed an analysis on c-BioPortal databases. As shown in Figure 7A, *ALDOA* had a high mutation frequency of 10% in lung adenocarcinoma (TCGA, PanCancer Atlas). The main genetic mutations of *ALDOA* were DNA copy number amplifications and mRNA upregulation (Figure 7B). Furthermore, compared with the unaltered group (n = 449), survival analysis revealed that the altered group (n = 56) was associated with poor overall survival (Figure 7C).

### Functional Enrichment Analysis of Positively Co-expressed Genes of *ALDOA*

**FIGURE 8:**
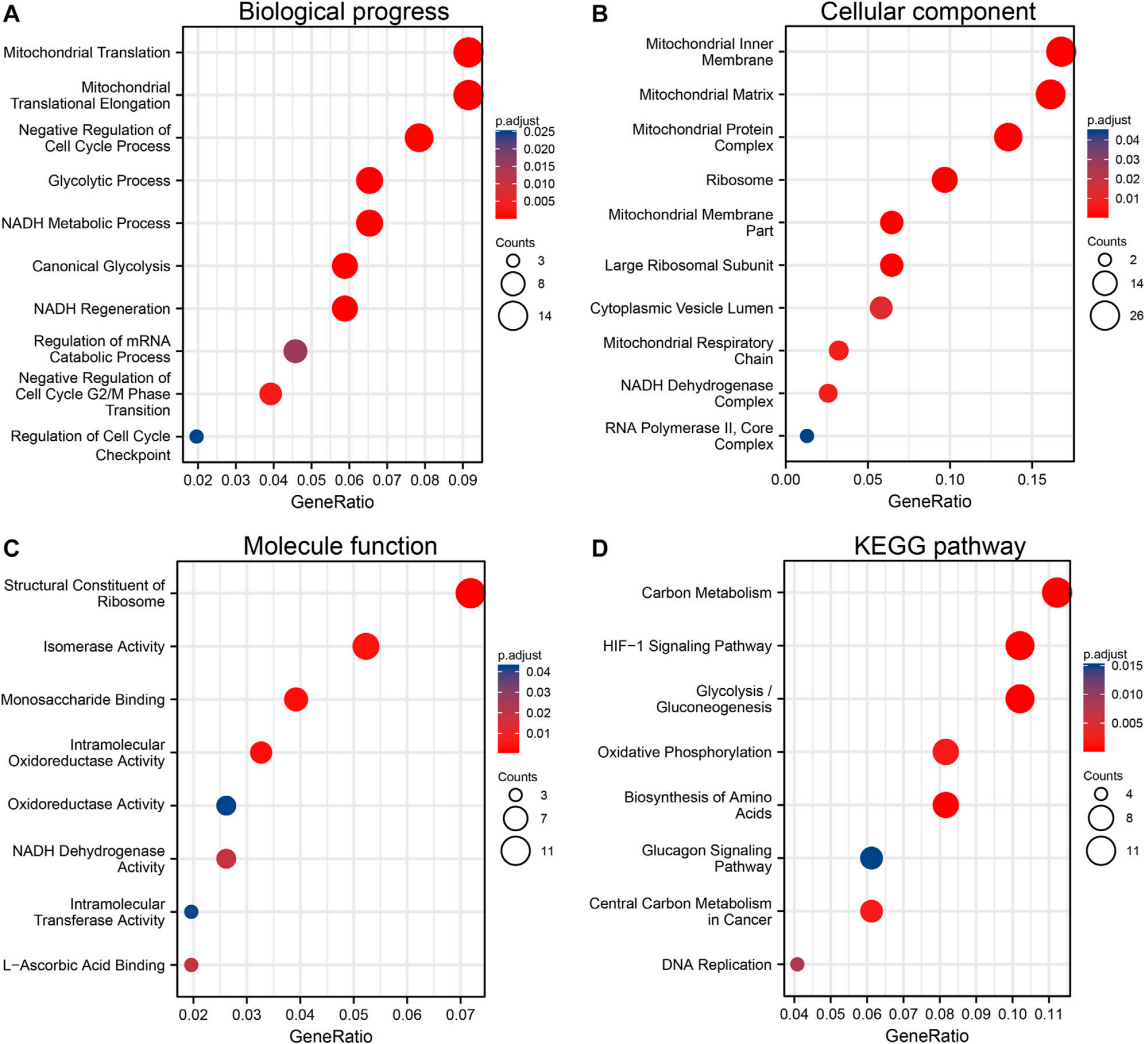
Functional enrichment analysis of positively co-expressed genes of *ALDOA*. **(A)** Biological progress of mitochondrial translation, mitochondrial translational elongation, and negative regulation of cell cycle progress **(B)** Cellular component of the mitochondrial inner membrane, mitochondrial matrix, and protein complex **(C)** Molecule function of the structural constituent of ribosome, isomerase activity, and monosaccharide binding **(D)** KEGG pathway analysis showed enrichment function in carbon metabolism, HIF-1 signaling pathway, and glycolysis/gluconeogenesis.

The top 300 co-expressed genes of *ALDOA* were downloaded from the c-BioPortal. As shown in Supplementary Table S1, *ALDOA* had 165 positively co-expressed genes. To further demonstrate the enrichment function of these positively co-expressed genes, we conducted the analyses of Gene Ontology (GO) and Kyoto Encyclopedia of Genes and Genomes (KEGG) pathway with R package clusterProfiler. GO analysis showed that positively co-expressed genes of *ALDOA* were involved in the biological progress of mitochondrial translation, mitochondrial translational elongation, and negative regulation of cell cycle progress (Figure 8A). They acted as structural constituents in the mitochondrial inner membrane, mitochondrial matrix, and protein complex (Figure 8B), and played an important part in the structural constituent of ribosome, isomerase activity, and monosaccharide binding (Figure 8C). KEGG pathway analysis in Figure 8D showed enrichment function in carbon metabolism, HIF-1 signaling pathway, and glycolysis/gluconeogenesis.

### Construction of PPI Networks, Identification, and Enrichment Function of Hub Genes Among Co-expressed Genes of *ALDOA*

**FIGURE 9:**
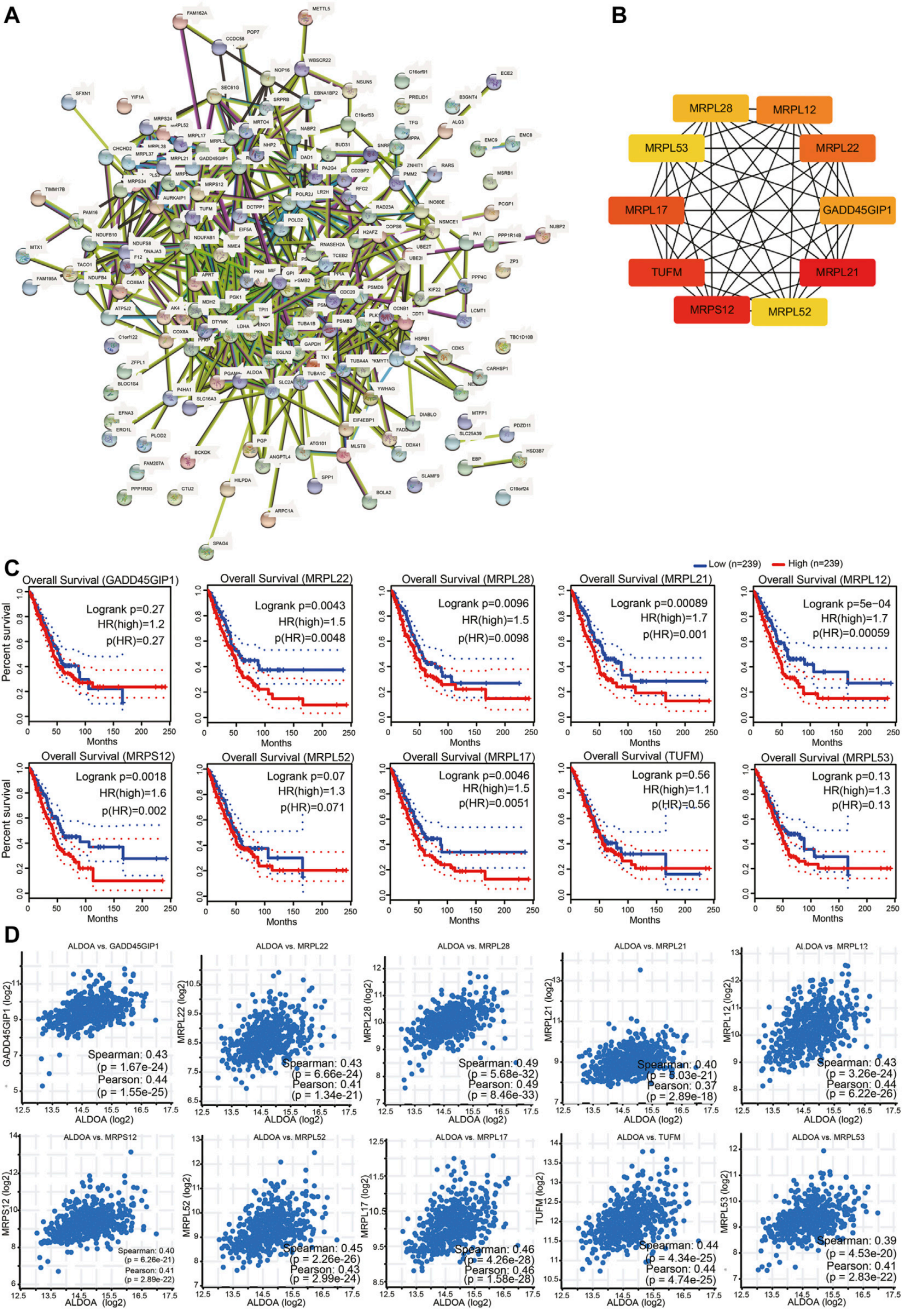
Construction of PPI networks and identification of hub genes among co-expressed genes of ALDOA **(A)** Construction of PPI networks by STRING with 165 positively co-expressed genes of *ALDOA* **(B)** Identification of ten hub genes of *ALDOA* with cytoHubba tool in Cytoscape **(C)** Prognostic importance analyses of 10 hub genes with GEPIA **(D)** Correlation between *ALDOA* and mRNA expression of 10 hub genes determined with c-BioPortal.

**FIGURE 10:**
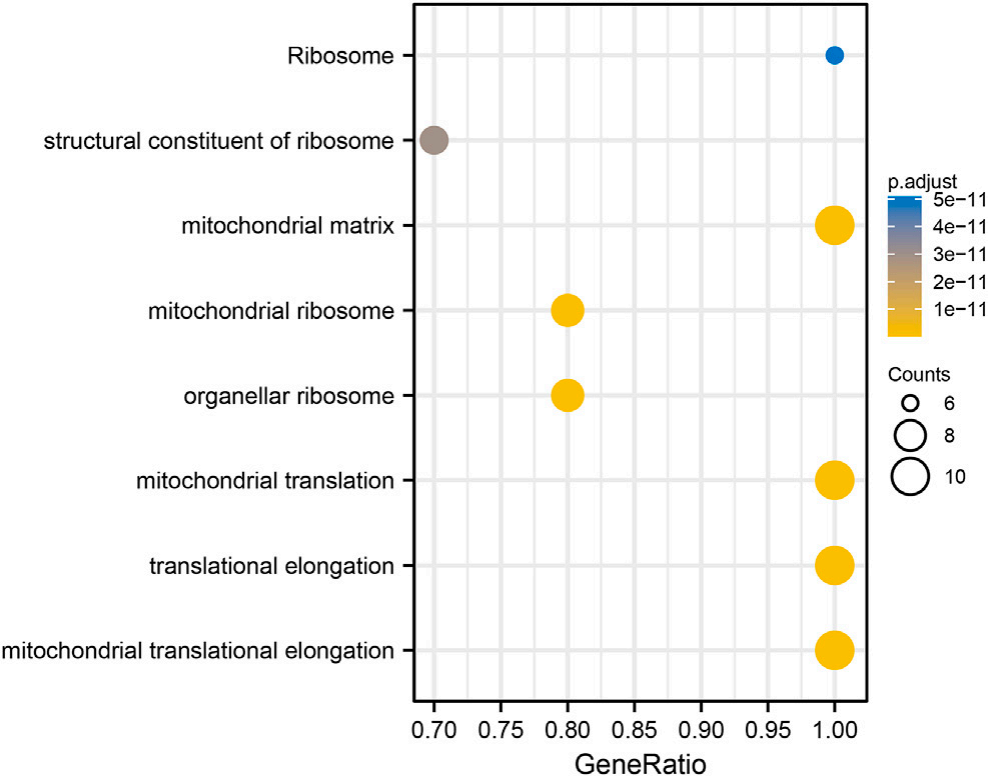
Functional enrichment analysis of the top 10 hub genes.

To construct PPI networks and identify hub genes of *ALDOA,* we imported the 165 positively co-expressed genes into STRING and then explored the degree scores with cytoHubba tool kits in Cytoscape. The PPI networks of co-expressed genes were shown in Figure 9A. As shown in Figure 9B, *GADD45GIP1*, *MRPL22*, *MRPL28*, *MRPL21*, *MRPL12*, *MRPS12*, *MRPL52*, *MRPL17*, *TUFM*, and *MRPL53* were the top 10 hub genes of *ALDOA.* To further evaluate their prognostic values and correlation with ALDOA in lung adenocarcinoma, we performed analyses on GEPIA and c-BioPortal. As shown in Figure 9C, upregulation of *MRPL22* (HR = 1.5, *p* = 0.0048), *MRPL28* (HR = 1.5, *p* = 0.0098), *MRPL21* (HR = 1.7, *p* = 0.001), *MRPL12* (HR = 1.7, *p* = 0.00059), *MRPS12* (HR = 1.6, *p* = 0.002), and *MRPL17* (HR = 1.5, *p* = 0.0051) were correlated with poor overall survival in lung adenocarcinoma. Based on the R-value of Spearman correlation were all more than 0.4, the genes were considered as the most potential hub genes of *ALDOA* (Figure 9C). We also conducted enrichment function of these top 10 hub genes with R package clusterProfiler. As shown in Figure 10, GO analysis showed they were involved in the biological progress of mitochondrial translational elongation, translational elongation, and mitochondrial translation. They may be associated with a molecular function of the structural constituent of the ribosome and acted as structural constituents in the organellar ribosome, mitochondrial ribosome, and mitochondrial matrix. KEGG pathway analysis showed enrichment function in the ribosome. Taken together, all these data suggest that these hub genes may play an important role in lung adenocarcinoma by cooperating with *ALDOA*.

### Relationship Between *ALDOA* Expression and Immune Infiltration in Lung Adenocarcinoma

**FIGURE 11:**
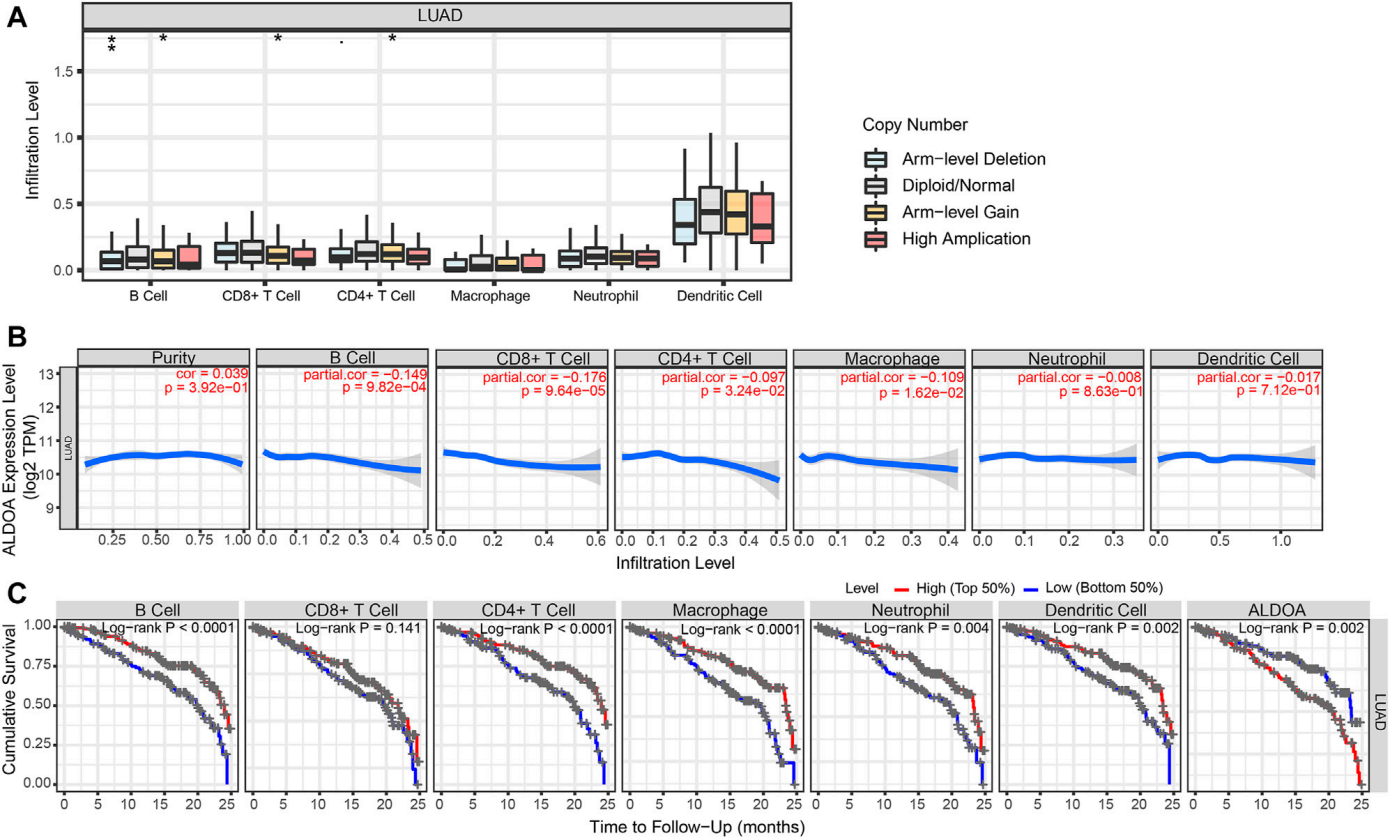
Immune cell infiltration of *ALDOA* in lung adenocarcinoma (**A**) The “SCNA” module analysis indicated that the copy number alterations of *ALDOA* were correlated with three immune cell infiltration levels **(B)** The “Gene” module analysis indicated that there was no correlation between *ALDOA* expression and tumor purity. However, *ALDOA* expression was negatively correlated with infiltrating levels of B cells, CD8^+^ T cells, CD4^+^ T cells, and macrophages (**C**) Low levels of B cells, CD4^+^ T cells, macrophages, neutrophils, and dendritic cells were associated with poor prognosis of lung adenocarcinoma patients.

To determine the potential relationship between *ALDOA* expression and immune infiltration levels in lung adenocarcinoma, we conducted a series of analyses by using TIMER. First, as shown in Figure 11A, the “SCNA” module analysis indicated that the copy number alterations of *ALDOA* were correlated with three immune cell infiltration levels, including B cells, CD8^+^ T cells, and CD4^+^ T cells in lung adenocarcinoma. Second, as shown in Figure 11B, the “Gene” module analysis indicated that there was no correlation between *ALDOA* expression and tumor purity. However, *ALDOA* expression was negatively correlated with infiltrating levels of B cells, CD8^+^ T cells, CD4^+^ T cells, and macrophages in lung adenocarcinoma. Third, to evaluate the impact of immune infiltration and *ALDOA* on the survival differences of lung adenocarcinoma patients, we used TIMER to draw Kaplan-Meier plots for immune infiltration and *ALDOA*. The results in Figure 11C showed that low levels of B cells, CD4^+^ T cells, macrophages, neutrophils, and dendritic cells were associated with poor prognosis of lung adenocarcinoma patients. On the contrary, high levels of *ALDOA* were correlated with the poor prognosis of lung adenocarcinoma patients. Taken together, these results suggest that *ALDOA* may regulate the expression level of tumor-infiltrating immune cells to affect lung adenocarcinoma and clinical prognosis.

### Correlation Between *ALDOA* Expression and Gene Markers of Tumor-Infiltrating Immune Cells

To further evaluate the relationship between *ALDOA* and tumor-infiltrating immune cells, we next explored the correlation between *ALDOA* expression and immunological markers in lung adenocarcinoma using the TIMER database. We determined *ALDOA* expression and immunological markers of various immune cells, including B cell, CD8^+^ T cell, T cell (general), M1 and M2 macrophage, neutrophils, and dendritic cell. After adjusting the correlation by tumor purity, these results revealed that there was a correlation between *ALDOA* expression and most immune marker sets (Table 3). In particular, *ALDOA* was significantly correlated with T cell markers (CD3E, CD2), neutrophils markers (CD66b, CCR7), and dendritic cell markers (HLA-DPB1, BDCA-1). We also assessed the correlation between *ALDOA* and these markers in lung adenocarcinoma using the GEPIA2 database, and the results were similar to those in TIMER (Supplementary Table S1).

**TABLE 3:** Correlation analysis between ALDOA and immune infiltration markers in TIMER.

Description	Gene markers	Lung adenocarcinoma			
		None		Purity	
		Cor	*p*	Cor	*p*
B cell	CD19	−0.157	**	0.039	3.92e-01
	CD79A	−0.106	1.59e-02	−0.104	2.04e-02
CD8^+^ T cell	CD8A	−0.133	*	−0.133	*
	CD8B	−0.150	**	−0.147	*
T cell (general)	CD3D	−0.130	*	−0.125	*
	CD3E	−0.169	**	−0.176	***
	CD2	−0.162	**	−0.166	**
M1 Macrophage	INOS (NOS2)	−0.012	7.79e-01	−0.010	8.24e-01
	IRF5	−0.023	6.08e-01	0.037	4.15e-01
	COX2 (PTGS2)	0.005	9.03e-01	−0.001	9.74e-01
M2 Macrophage	CD163	−0.030	5.03e-01	−0.004	9.22e-01
	VSIG4	−0.042	3.36e-01	−0.028	5.38e-01
	MS4A4A	−0.154	**	0.039	3.92e-01
Neutrophils	CD66b (CEACAM8)	−0.180	***	−0.183	**
	CD11b (ITGAM)	0.023	6.06e-01	0.123	3.00e-01
	CCR7	−0.199	***	−0.203	***
Dendritic cell	HLA-DPB1	−0.164	**	−0.156	**
	HLA-DQB1	−0.101	2.15e-02	−0.085	5.84e-02
	HLA-DRA	−0.157	**	−-0.144	*
	HLA-DPA1	−0.122	*	−0.111	1.34e-02
	BDCA-1 (CD1C)	−0.249	***	−0.240	***
	BDCA-4 (NRP1)	0.033	4.59e-01	0.033	4.59e-01
	CD11c (ITGAX)	−0.002	9.69e-01	0.015	7.33e-01

Cor, correlation of Spearman’s R value; None, correlation with no adjustment; Purity, correlation with adjusted by purity.

*p* < 0.01; *p* < 0.001; *p* < 0.0001.

## Discussion

Many studies about the dysregulation of the *ALDOA* gene have emerged in recent years, including colorectal cancer (Dai et al., 2018), gastric cancer (Jiang et al., 2018), and renal cell carcinoma (Huang et al., 2018). Previous bioinformatics results also indicated that *ALDOA* expression was correlated with prognosis in bladder cancer (Li et al., 2019), hepatocellular cancer (Tang et al., 2021). In lung cancer, overexpression of *ALDOA* is reported to promote lung cancer cell proliferation and metastasis (Chang et al., 2019). Moreover, Zhang *et al.* reported that upregulated transcriptional levels of *ALDOA* were correlated with cell cycle-related genes and could regulate progress in non-small cell lung cancer (Zhang et al., 2017). However, the relationship between the expression level of *ALDOA* and prognostic value and immune infiltration of lung adenocarcinoma has not been studied. To the best of our knowledge, for the first time, our study explored the prognostic value and correlation with immune infiltration of *ALDOA* in lung adenocarcinoma.

In this study, based on the data from Oncomine, TCGA, UALCAN, and HPA, we revealed that the mRNA and protein expression of *ALDOA* is upregulated in lung adenocarcinoma tissues. Given that there are significant differences between lung adenocarcinoma and normal tissues grouped by T stage, N stage, M stage, and TNM stage, we conclude that *ALDOA* might promote tumorigenesis and metastasis in lung adenocarcinoma. A paper from Marcišauskas *et al.* reported that *ALDOA* in cyst fluids and serum can be used as a diagnostic biomarker to separate stage I type 1 and type 2 ovarian cancers from benign serous adenoma (Marcišauskas et al., 2019). ROC curve can be used to examine the diagnostic value of biomarkers (Do and Le, 2021; Le et al., 2021). In the current study, ROC curve analysis suggested that *ALDOA* can act as a prospective non-invasive diagnostic biomarker to differentiate lung adenocarcinoma tissues from adjacent normal tissues. Previous studies reported that upregulation of *ALDOA* is correlated with poor prognosis in colorectal cancer (Dai et al., 2018), gastric cancer (Jiang et al., 2018), and hepatocellular carcinoma (Tang et al., 2021). Our data on survival analysis with GEPIA2 and the Kaplan Meier plotter indicated that lung adenocarcinoma patients with high *ALDOA* expression or genetic alteration have a poor overall survival prognosis. Univariate and multivariate analysis revealed that *ALDOA* is an independent poor prognostic factor for overall survival in lung adenocarcinoma. The major strength of this study is our findings raise the possibility that the upregulation of *ALDOA* could be a potential prognostic marker in lung adenocarcinoma.

Functional enrichment analysis was carried out to further explore the role of these positively co-expressed genes with *ALDOA* in lung adenocarcinoma. GO enrichment analysis showed that these positively co-expressed genes of *ALDOA* were involved in the biological progress of mitochondrial translation and negative regulation of cell cycle progression. KEGG pathway enrichment analysis showed enrichment function in carbon metabolism, HIF-1 signaling pathway, and glycolysis/gluconeogenesis. It is well known that the HIF-1 signaling pathway and glycolysis/gluconeogenesis play an important role in tumor invasion and metastasis (Chen et al., 2011; Rankin and Giaccia, 2016; Peng et al., 2020). Based on our results, we speculate that *ALDOA* may be involved in the progress of invasion and metastasis in lung adenocarcinoma. However, this should be tested in other experiments. Moreover, we also imported the positively co-expressed genes of *ALDOA* into the STRING database and Cytoscape to obtain the PPI network and identify hub genes. In light of STRING database analysis, we conducted a PPI network and interactions among these positively co-expressed genes. The Cytoscape with cytoHubba tool kits, GEPIA, and c-BioPortal analysis suggested that upregulated hub genes of *MRPL22*, *MRPL28*, *MRPL21*, *MRPL12*, *MRPS12*, and *MRPL17* are correlated with poor overall survival and may play a key role by cooperating with *ALDOA* in lung adenocarcinoma.

Many studies about the possible role of immune infiltration have emerged in recent years. It is reported that immune infiltration is correlated with prognosis in human tumors (Pagès et al., 2010; Lei et al., 2020). However, the relationship between *ALDOA* expression and immune infiltration has not been investigated. In the present study, we reported that *ALDOA* copy number alterations were correlated with immune infiltration levels of B cells, CD8^+^ T cells, and CD4^+^ T cells in lung adenocarcinoma by TIMER. We also confirmed that *ALDOA* gene expression was inversely correlated with infiltrating levels of B cells, CD8^+^ T cells, CD4^+^ T cells, and macrophages in lung adenocarcinoma. Moreover, previous studies showed that there was a correlation between tumor-infiltrating immune cell expression and the prognosis of lung cancer patients (Liu et al., 2017; Wang et al., 2019; Pan et al., 2020). In this study, our results showed low levels of B cells, CD4^+^ T cells, macrophages, neutrophils, and dendritic cells were associated with poor prognosis of lung adenocarcinoma patients, while high *ALDOA* expression was correlated with poor prognosis in lung adenocarcinoma patients. Based on our data, we conclude for the first time that *ALDOA* is correlated with immune infiltration in lung adenocarcinoma. We further speculate that *ALDOA* can regulate the expression level of tumor-infiltrating immune cells to affect the clinical prognosis of lung adenocarcinoma patients.

In conclusion, our research suggests that the upregulation of *ALDOA* is correlated with tumorigenesis and metastasis in lung adenocarcinoma. Our results show high expression of *ALDOA* predicts poor prognosis and *ALDOA* is an independent poor prognostic factor for overall survival. *ALDOA* may regulate tumor-infiltrating immune cells to affect the clinical prognosis of lung adenocarcinoma patients. Our data provide a potential prognostic biomarker and therapeutic target for lung adenocarcinoma.

## Data Availability

The original contributions presented in the study are included in the article/Supplementary Material, further inquiries can be directed to the corresponding author.
