# Supplementary material for: Prognostic Implications and Immune Infiltration Analysis of ALDOA in Lung Adenocarcinoma
Source: Front Genet. 2021 Dec 3;12:721021. doi: 10.3389/fgene.2021.721021 (PMC8678114; doi:10.3389/fgene.2021.721021)
Supplement: Supplementary file 2 [file Table1.docx]

**Supplementary Table 1** The top 165 positively co-expressed genes of ALDOA

| Correlated Gene | Cytoband | Spearman's Correlation | p-Value | q-Value |
| --- | --- | --- | --- | --- |
| GAPDH | 12p13.31 | 0.66 | 4.42E-65 | 8.89E-61 |
| PPP4C | 16p11.2 | 0.638 | 1.57E-59 | 1.58E-55 |
| PKM | 15q23 | 0.581 | 1.87E-47 | 1.26E-43 |
| TPI1 | 12p13.31 | 0.574 | 4.69E-46 | 2.36E-42 |
| FAM162A | 3q21.1 | 0.569 | 4.50E-45 | 1.81E-41 |
| ENO1 | 1p36.23 | 0.564 | 3.34E-44 | 1.12E-40 |
| LDHA | 11p15.1 | 0.546 | 5.91E-41 | 1.70E-37 |
| BOLA2 | 16p11.2 | 0.542 | 2.98E-40 | 7.50E-37 |
| GPI | 19q13.11 | 0.536 | 3.24E-39 | 7.25E-36 |
| PGK1 | Xq21.1 | 0.522 | 6.42E-37 | 1.29E-33 |
| NDUFAB1 | 16p12.2 | 0.507 | 9.92E-35 | 1.82E-31 |
| MIF | 22q11.23 | 0.503 | 4.69E-34 | 7.87E-31 |
| SPAG4 | 20q11.22 | 0.501 | 1.05E-33 | 1.62E-30 |
| PLOD2 | 3q24 | 0.497 | 4.08E-33 | 5.48E-30 |
| SLC2A1 | 1p34.2 | 0.492 | 1.85E-32 | 2.33E-29 |
| NDUFB4 | 3q13.33 | 0.491 | 2.84E-32 | 3.36E-29 |
| MRPL28 | 16p13.3 | 0.489 | 5.68E-32 | 6.35E-29 |
| P4HA1 | 10q22.1 | 0.483 | 3.84E-31 | 4.07E-28 |
| MRPS34 | 16p13.3 | 0.479 | 1.40E-30 | 1.34E-27 |
| ALG3 | 3q27.1 | 0.478 | 2.03E-30 | 1.86E-27 |
| MSRB1 | 16p13.3 | 0.477 | 2.84E-30 | 2.38E-27 |
| PRELID1 | 5q35.3 | 0.477 | 2.84E-30 | 2.38E-27 |
| RAB5IF | 20q11.23 | 0.472 | 1.14E-29 | 9.18E-27 |
| SLC16A3 | 17q25.3 | 0.469 | 2.90E-29 | 2.17E-26 |
| C16ORF91 | 16p13.3 | 0.469 | 3.43E-29 | 2.46E-26 |
| INO80E | 16p11.2 | 0.468 | 4.41E-29 | 3.06E-26 |
| PGAM1 | 10q24.1 | 0.465 | 1.06E-28 | 6.90E-26 |
| ELOB | 16p13.3 | 0.463 | 2.12E-28 | 1.33E-25 |
| TK1 | 17q25.3 | 0.462 | 2.20E-28 | 1.34E-25 |
| HSD3B7 | 16p11.2 | 0.461 | 3.20E-28 | 1.89E-25 |
| MRPL17 | 11p15.4 | 0.46 | 4.26E-28 | 2.38E-25 |
| TBC1D10B | 16p11.2 | 0.458 | 7.28E-28 | 3.96E-25 |
| POP7 | 7q22.1 | 0.451 | 6.67E-27 | 3.05E-24 |
| DNAJA3 | 16p13.3 | 0.45 | 8.49E-27 | 3.80E-24 |
| ERO1A | 14q22.1 | 0.449 | 1.27E-26 | 5.44E-24 |
| ANGPTL4 | 19p13.2 | 0.448 | 1.52E-26 | 6.12E-24 |
| MRPL52 | 14q11.2 | 0.447 | 2.26E-26 | 8.90E-24 |
| PSMB3 | 17q12 | 0.445 | 3.55E-26 | 1.37E-23 |
| NDUFB10 | 16p13.3 | 0.445 | 3.98E-26 | 1.51E-23 |
| CCDC58 | 3q21.1 | 0.444 | 4.33E-26 | 1.58E-23 |
| COPS6 | 7q22.1 | 0.444 | 4.90E-26 | 1.76E-23 |
| BCKDK | 16p11.2 | 0.44 | 1.30E-25 | 4.52E-23 |
| PFKP | 10p15.2 | 0.44 | 1.35E-25 | 4.61E-23 |
| MDH2 | 7q11.23 | 0.438 | 2.37E-25 | 7.56E-23 |
| DCTPP1 | 16p11.2 | 0.437 | 3.28E-25 | 1.02E-22 |
| RPL26L1 | 5q35.1 | 0.437 | 3.42E-25 | 1.04E-22 |
| TUFM | 16p11.2 | 0.436 | 4.34E-25 | 1.27E-22 |
| F12 | 5q35.3 | 0.436 | 4.37E-25 | 1.27E-22 |
| EBNA1BP2 | 1p34.2 | 0.436 | 4.96E-25 | 1.42E-22 |
| FADD | 11q13.3 | 0.435 | 5.18E-25 | 1.45E-22 |
| HSPB1P1 | 9q21.13 | 0.434 | 6.72E-25 | 1.85E-22 |
| PAM16 | 16p13.3 | 0.433 | 9.48E-25 | 2.51E-22 |
| TUBA4A | 2q35 | 0.433 | 1.01E-24 | 2.63E-22 |
| DTYMK | 2q37.3 | 0.433 | 1.09E-24 | 2.82E-22 |
| PMM2 | 16p13.2 | 0.433 | 1.12E-24 | 2.86E-22 |
| DIABLO | 12q24.31 | 0.432 | 1.16E-24 | 2.93E-22 |
| EMC9 | 14q12 | 0.431 | 1.61E-24 | 4.00E-22 |
| GADD45GIP1 | 19p13.13 | 0.431 | 1.67E-24 | 4.11E-22 |
| ECE2 | 3q27.1 | 0.431 | 1.85E-24 | 4.42E-22 |
| PPP1R3G | 6p25.1 | 0.431 | 1.87E-24 | 4.42E-22 |
| RNASEH2A | 19p13.13 | 0.43 | 2.09E-24 | 4.90E-22 |
| PSMB2 | 1p34.3 | 0.43 | 2.16E-24 | 5.00E-22 |
| TUBA1C | 12q13.12 | 0.43 | 2.37E-24 | 5.42E-22 |
| MTX1 | 1q22 | 0.43 | 2.44E-24 | 5.50E-22 |
| AK4 | 1p31.3 | 0.43 | 2.46E-24 | 5.50E-22 |
| NOP16 | 5q35.2 | 0.429 | 3.13E-24 | 6.92E-22 |
| MRPL12 | 17q25.3 | 0.429 | 3.26E-24 | 7.13E-22 |
| PKMYT1 | 16p13.3 | 0.428 | 4.06E-24 | 8.51E-22 |
| MTFP1 | 22q12.2 | 0.427 | 4.81E-24 | 9.87E-22 |
| CHCHD2 | 7p11.2 | 0.427 | 5.31E-24 | 1.08E-21 |
| MRPL22 | 5q33.2 | 0.426 | 6.66E-24 | 1.29E-21 |
| MRPS24 | 7p13 | 0.425 | 8.96E-24 | 1.72E-21 |
| CARHSP1 | 16p13.2 | 0.424 | 1.16E-23 | 2.14E-21 |
| TFG | 3q12.2 | 0.423 | 1.49E-23 | 2.68E-21 |
| HSPB1 | 7q11.23 | 0.423 | 1.63E-23 | 2.88E-21 |
| KIF22 | 16p11.2 | 0.419 | 4.73E-23 | 8.07E-21 |
| C19ORF53 | 19p13.13 | 0.418 | 6.02E-23 | 1.01E-20 |
| PLK1 | 16p12.2 | 0.417 | 6.49E-23 | 1.07E-20 |
| AURKAIP1 | 1p36.33 | 0.417 | 7.90E-23 | 1.27E-20 |
| C1ORF122 | 1p34.3 | 0.416 | 1.00E-22 | 1.57E-20 |
| EGLN3 | 14q13.1 | 0.416 | 1.00E-22 | 1.57E-20 |
| NSMCE1 | 16p12.1 | 0.416 | 1.01E-22 | 1.57E-20 |
| ATG101 | 12q13.13 | 0.415 | 1.07E-22 | 1.66E-20 |
| SFXN1 | 5q35.2 | 0.415 | 1.17E-22 | 1.79E-20 |
| PGP | 16p13.3 | 0.415 | 1.31E-22 | 1.98E-20 |
| BUD31 | 7q22.1 | 0.415 | 1.33E-22 | 1.99E-20 |
| SSBP1 | 7q34 | 0.414 | 1.53E-22 | 2.27E-20 |
| DDX41 | 5q35.3 | 0.413 | 2.01E-22 | 2.91E-20 |
| EBP | Xp11.23 | 0.413 | 2.19E-22 | 3.15E-20 |
| PPP1R14B | 11q13.1 | 0.412 | 2.85E-22 | 4.07E-20 |
| ATP5MF | 7q22.1 | 0.411 | 2.97E-22 | 4.21E-20 |
| NDUFS8 | 11q13.2 | 0.411 | 3.61E-22 | 5.05E-20 |
| PDZD11 | Xq13.1 | 0.41 | 4.49E-22 | 6.15E-20 |
| RARS | 5q34 | 0.409 | 5.10E-22 | 6.89E-20 |
| MRPL37 | 1p32.3 | 0.409 | 5.99E-22 | 7.98E-20 |
| POLR2J | 7q22.1 | 0.407 | 8.97E-22 | 1.16E-19 |
| POLD2 | 7p13 | 0.407 | 1.02E-21 | 1.30E-19 |
| RAD23A | 19p13.13 | 0.406 | 1.19E-21 | 1.48E-19 |
| FAM207A | 21q22.3 | 0.406 | 1.25E-21 | 1.54E-19 |
| RFC2 | 7q11.23 | 0.405 | 1.32E-21 | 1.62E-19 |
| UBE2I | 16p13.3 | 0.405 | 1.53E-21 | 1.84E-19 |
| APRT | 16q24.3 | 0.405 | 1.61E-21 | 1.92E-19 |
| NABP2 | 12q13.3 | 0.405 | 1.62E-21 | 1.92E-19 |
| PPIA | 7p13 | 0.405 | 1.63E-21 | 1.93E-19 |
| HILPDA | 7q32.1 | 0.404 | 1.70E-21 | 2.01E-19 |
| SRPRB | 3q22.1 | 0.403 | 2.45E-21 | 2.82E-19 |
| CYTOR | 2p11.2 | 0.402 | 3.21E-21 | 3.60E-19 |
| SEC61G | 7p11.2 | 0.402 | 3.41E-21 | 3.81E-19 |
| ZNHIT1 | 7q22.1 | 0.401 | 3.83E-21 | 4.22E-19 |
| PSMB5 | 14q11.2 | 0.4 | 4.89E-21 | 5.26E-19 |
| MRPL21 | 11q13.3 | 0.4 | 5.03E-21 | 5.36E-19 |
| H2AFZ | 4q23 | 0.4 | 5.03E-21 | 5.36E-19 |
| EIF5A | 17p13.1 | 0.4 | 5.50E-21 | 5.76E-19 |
| YIF1A | 11q13.2 | 0.399 | 5.83E-21 | 6.08E-19 |
| CDK5 | 7q36.1 | 0.399 | 5.91E-21 | 6.13E-19 |
| LCMT1 | 16p12.1 | 0.399 | 5.96E-21 | 6.16E-19 |
| PA2G4 | 12q13.2 | 0.399 | 6.19E-21 | 6.36E-19 |
| MRPS12 | 19q13.2 | 0.399 | 6.26E-21 | 6.40E-19 |
| POLR2H | 3q27.1 | 0.399 | 6.68E-21 | 6.76E-19 |
| COX17 | 3q13.33 | 0.399 | 7.28E-21 | 7.24E-19 |
| BLOC1S4 | 4p16.1 | 0.398 | 7.84E-21 | 7.69E-19 |
| NEIL3 | 4q34.3 | 0.398 | 8.19E-21 | 8.00E-19 |
| TRAP1 | 16p13.3 | 0.397 | 9.55E-21 | 9.19E-19 |
| EMC8 | 16q24.1 | 0.397 | 9.68E-21 | 9.28E-19 |
| METTL5 | 2q31.1 | 0.397 | 1.09E-20 | 1.03E-18 |
| MLST8 | 16p13.3 | 0.396 | 1.21E-20 | 1.13E-18 |
| PSMD9 | 12q24.31 | 0.396 | 1.27E-20 | 1.19E-18 |
| UBE2T | 1q32.1 | 0.396 | 1.32E-20 | 1.23E-18 |
| NME4 | 16p13.3 | 0.396 | 1.40E-20 | 1.28E-18 |
| SLAMF9 | 1q23.2 | 0.396 | 1.41E-20 | 1.28E-18 |
| NHP2 | 5q35.3 | 0.395 | 1.60E-20 | 1.43E-18 |
| NSUN5 | 7q11.23 | 0.395 | 1.66E-20 | 1.48E-18 |
| MRTO4 | 1p36.13 | 0.394 | 2.11E-20 | 1.85E-18 |
| EIF4EBP1 | 8p11.23 | 0.394 | 2.27E-20 | 1.99E-18 |
| MCRIP2 | 16p13.3 | 0.394 | 2.32E-20 | 2.03E-18 |
| CDC20 | 1p34.2 | 0.393 | 2.65E-20 | 2.25E-18 |
| CCNB1 | 5q13.2 | 0.393 | 2.98E-20 | 2.52E-18 |
| TIMM17B | Xp11.23 | 0.392 | 3.32E-20 | 2.77E-18 |
| COX8A | 11q13.1 | 0.392 | 3.69E-20 | 3.02E-18 |
| DAD1 | 14q11.2 | 0.392 | 3.80E-20 | 3.08E-18 |
| GMPPA | 2q35 | 0.391 | 4.46E-20 | 3.60E-18 |
| MRPL53 | 2p13.1 | 0.391 | 4.53E-20 | 3.65E-18 |
| ARPC1A | 7q22.1 | 0.391 | 4.93E-20 | 3.95E-18 |
| TUBA1B | 12q13.12 | 0.39 | 5.70E-20 | 4.46E-18 |
| PCGF1 | 2p13.1 | 0.39 | 5.75E-20 | 4.49E-18 |
| CD2BP2 | 16p11.2 | 0.39 | 6.15E-20 | 4.76E-18 |
| YWHAG | 7q11.23 | 0.39 | 6.24E-20 | 4.81E-18 |
| CDT1 | 16q24.3 | 0.389 | 6.66E-20 | 5.11E-18 |
| ZFPL1 | 11q13.1 | 0.389 | 7.10E-20 | 5.43E-18 |
| PAGR1 | 16p11.2 | 0.389 | 7.27E-20 | 5.55E-18 |
| B3GNT4 | 12q24.31 | 0.389 | 7.90E-20 | 5.98E-18 |
| EFNA3 | 1q21.3 | 0.388 | 8.42E-20 | 6.35E-18 |
| LOC388955 | 2p15 | 0.388 | 8.62E-20 | 6.48E-18 |
| BUD23 | 7q11.23 | 0.388 | 8.86E-20 | 6.63E-18 |
| SNRPA1 | 15q26.3 | 0.387 | 1.04E-19 | 7.66E-18 |
| ZP3 | 7q11.23 | 0.387 | 1.07E-19 | 7.85E-18 |
| SPP1 | 4q22.1 | 0.387 | 1.09E-19 | 7.92E-18 |
| NUBP2 | 16p13.3 | 0.387 | 1.20E-19 | 8.61E-18 |
| C19ORF24 | 19p13.3 | 0.387 | 1.21E-19 | 8.69E-18 |
| HMMR | 5q34 | 0.387 | 1.27E-19 | 9.08E-18 |
| SLC25A39 | 17q21.31 | 0.386 | 1.36E-19 | 9.64E-18 |
| CTU2 | 16q24.3 | 0.386 | 1.48E-19 | 1.04E-17 |
| TACO1 | 17q23.3 | 0.385 | 1.71E-19 | 1.19E-17 |
| COX6A1 | 12q24.31\|12q24.2 | 0.385 | 1.77E-19 | 1.21E-17 |
| PSMA5 | 1p13.3 | 0.384 | 2.11E-19 | 1.42E-17 |
