# Supplementary material for: Prognostic Implications and Immune Infiltration Analysis of ALDOA in Lung Adenocarcinoma
Source: Front Genet. 2021 Dec 3;12:721021. doi: 10.3389/fgene.2021.721021 (PMC8678114; doi:10.3389/fgene.2021.721021)
Supplement: Supplementary file 4 [file Table2.docx]

**Supplementary Table 2** Correlation analysis between ALDOA and immune infiltration markers from GEPIA.

| **Description** | **Gene markers** | **Lung adenocarcinoma** | | | |
| --- | --- | --- | --- | --- | --- |
|  |  | **Tumor** | | **Normal** | |
|  |  | **Cor** | ***P*** | **Cor** | ***P*** |
| B cell | CD19 | -0.21 | 4.8e-06 | 0.097 | 0.47 |
|  | CD79A | -0.20 | 1.4e-05 | 0.084 | 0.53 |
| T cell (general) | CD3D | -0.17 | 1.9e-04 | -0.19 | 0.15 |
|  | CD3E | -0.21 | 3.0e-06 | -0.11 | 0.42 |
|  | CD2 | -0.19 | 2.1e-05 | -0.3 | 0.019 |
| CD8+ T cell | CD8A | -0.18 | 8.7e-05 | 0.0021 | 0.99 |
|  | CD8B | -0.19 | 1.7e-05 | -0.05 | 0.71 |
| M1 Macrophage | INOS (NOS2) | -0.024 | 0.6 | -0.081 | 0.54 |
|  | IRF5 | 0.011 | 0.8 | 0.66 | 1.2e-08 |
|  | COX2 (PTGS2) | 0.017 | 0.7 | -0.12 | 0.36 |
| M2 Macrophage | CD163 | -0.059 | 0.19 | 0.66 | 1.3e-08 |
|  | VSIG4 | -0.072 | 0.12 | 0.65 | 2.5e-08 |
|  | MS4A4A | -0.19 | 2.3e-05 | 0.58 | 1.3e-06 |
| Neutrophils | CD66b (CEACAM8) | -0.16 | 4e-04 | 0.23 | 0.081 |
|  | CD11b (ITGAM) | -0.028 | 0.54 | 0.57 | 3.1e-06 |
|  | CCR7 | -0.22 | 8.7e-07 | 0.093 | 0.49 |
| Dendritic cell | HLA-DPB1 | -0.17 | 1.3e-04 | 0.3 | 0.02 |
|  | HLA-DQB1 | -0.063 | 0.17 | -0.033 | 0.8 |
|  | HLA-DRA | -0.19 | 3.1e-05 | 0.3 | 0.02 |
|  | HLA-DPA1 | -0.15 | 0.001 | 0.23 | 0.079 |
|  | BDCA-1 (CD1C) | -0.23 | 5.8e-07 | -0.085 | 0.52 |
|  | BDCA-4 (NRP1) | 0.029 | 0.52 | -0.043 | 0.75 |
|  | CD11c (ITGAX) | -0.032 | 0.48 | 0.58 | 1.2e-06 |

Cor. correlation of Spearman’s R value; * < P 0.01; **P < 0.001;***P < 0.0001.
